# Fluorodeoxyglucose (FDG) Uptake in the Remnant Adrenal Gland Mimicking Tumor Recurrence in a Patient With Adrenocortical Carcinoma After Treatment With Mitotane

**DOI:** 10.7759/cureus.55486

**Published:** 2024-03-04

**Authors:** Juan D Ayala Torres, Brian Noreña Rengifo

**Affiliations:** 1 Radiology, Universidad de Antioquia, Medellín, COL

**Keywords:** radiology, adrenocortical carcinoma, diagnostic errors, mitotane, positron emission tomography computed tomography

## Abstract

Adrenocortical carcinoma (ACC) is a rare malignancy with poor prognosis. Its diagnosis requires clinical suspicion and confirmation through laboratory and imaging studies, including computed tomography (CT), magnetic resonance imaging (MRI), and abdominal ultrasound, as well as histological confirmation. Positron emission tomography (PET) is useful for distinguishing between benign and malignant lesions and for evaluating tumor recurrences or metastases. A case is described in which the uptake of fluorodeoxyglucose (18F-FDG) in a remnant adrenal gland could be misinterpreted as tumoral pathology. The article presents the case of a patient with ACC who, after treatment, showed increased FDG uptake in the remnant adrenal gland, which disappeared after discontinuation of treatment with mitotane. Possible explanations for this increase in FDG uptake are discussed, including the action of mitotane. In summary, it is highlighted that FDG uptake in remnant adrenal glands in patients treated with mitotane does not always indicate tumor recurrence or adrenal hypertrophy.

## Introduction

Adrenocortical carcinoma (ACC) is a rare malignancy with a poor prognosis [1,2]. Diagnosis requires clinical suspicion, laboratory and imaging studies such as computed tomography (CT), magnetic resonance imaging (MRI), and abdominal ultrasound, and histological confirmation [2-4]. Positron emission tomography (PET) is a useful diagnostic method in differentiating between benign and malignant lesions; it is also indicated in the evaluation of tumor recurrences or metastases in patients with known malignancies [2-4]. The aim of this article is to describe a case in which the uptake of fluorodeoxyglucose (18F-FDG) in the remnant adrenal gland of a patient with a history of adrenocortical carcinoma could be misinterpreted as tumoral pathology.

## Case presentation

**Figure 1:**
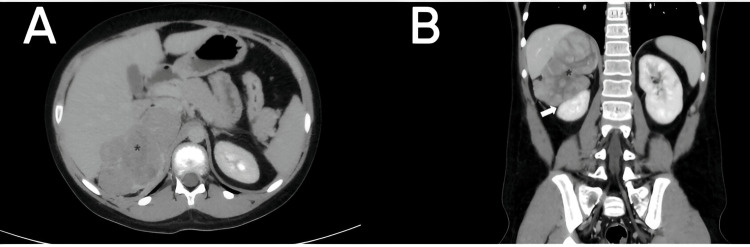
Abdominal computed tomography with intravenous contrast in axial (A) and coronal (B) planes. Mass with heterogeneous enhancement and large size in the right adrenal gland (asterisk in A and B), with loss of cleavage plane with the right lobe of the liver and displacing the kidney downward (arrow in B).

We report the case of an 11-year-old male patient referred to our institution due to the presence of a right adrenal mass. Initially, a contrast-enhanced CT scan was performed, confirming a large lesion with heterogeneous enhancement with contrast medium in the right adrenal gland (Figure 1).

**Figure 2:**
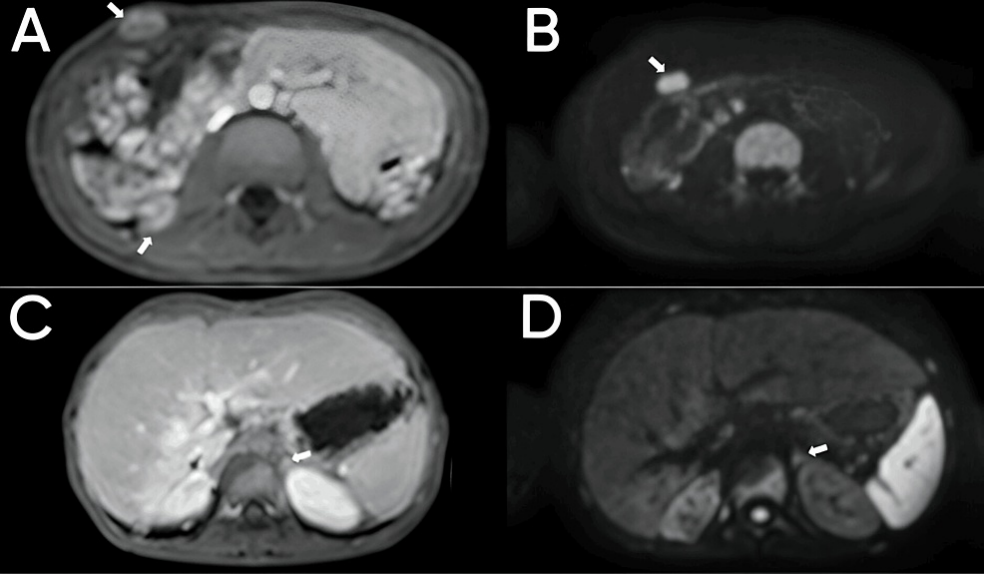
Abdominal magnetic resonance imaging with intravenous contrast in the axial plane following contrast administration (A and C) and diffusion-weighted sequence with B 800 (B and D). Nodular lesions in the abdominal wall and the right psoas muscle enhancing with contrast medium (arrows in A) and restricting diffusion (arrows in B) consistent with tumoral implants. The left adrenal gland appears normal in size and enhancement with contrast medium (arrow in C), without diffusion restriction foci (arrow in D).

Right adrenalectomy with lymphadenectomy was performed by laparotomy, and histological analysis confirmed the diagnosis of ACC. Subsequently, the patient received local radiotherapy and adjuvant treatment with mitotane. Nine months after surgery, contrast-enhanced abdominal MRI demonstrated tumor lesions in the right psoas and abdominal wall, with the left adrenal gland showing normal morphology (Figure 2).

**Figure 3:**
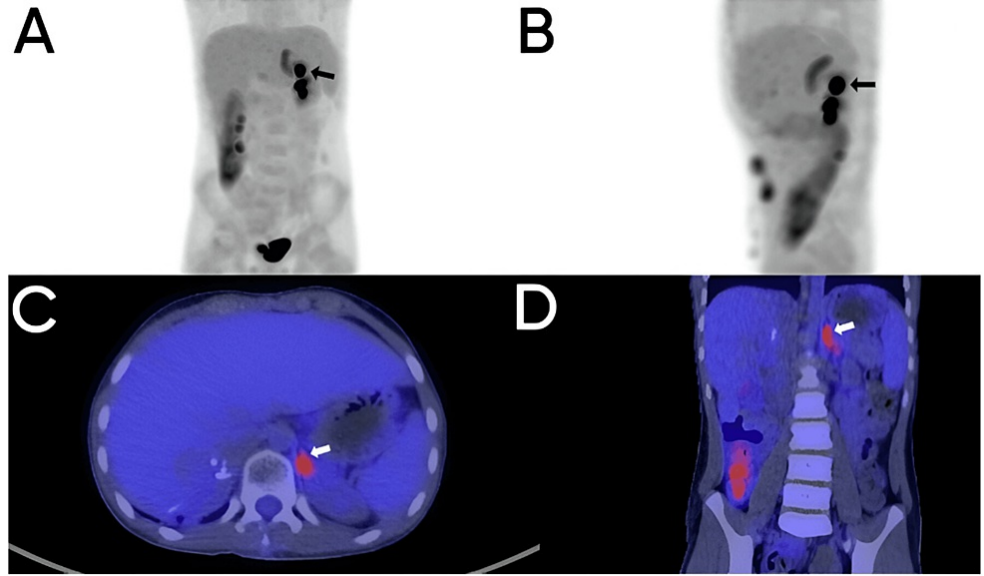
Positron emission tomography with fluorodeoxyglucose (PET-FDG). Maximum intensity projection (MIP) volumetric images of PET with FDG (A and B), fusion of plain computed tomography and PET-FDG in the axial plane (C) and coronal plane (D). Focal FDG uptake in the left adrenal gland is evident in the MIP volumetric images of PET with FDG (A and B). In the fusion of CT and PET-FDG, it is confirmed that the focus of increased uptake corresponds to the left adrenal gland with a maximum SUV of 3.95 (arrow in C and D).

Eleven months post-surgery, positron emission tomography with CT (PET/CT) using 18F-FDG was performed to assess the treatment response, identifying increased uptake in known tumor implants with a maximum standardized uptake value (SUVmax) of 4.88 and 4.04, respectively, indicating tumoral viability. Additionally, diffuse uptake was observed in the left adrenal gland with an SUVmax of 3.95, which on corresponding CT did not correlate with focal lesions or increased gland size (Figure 3).

**Figure 4:**
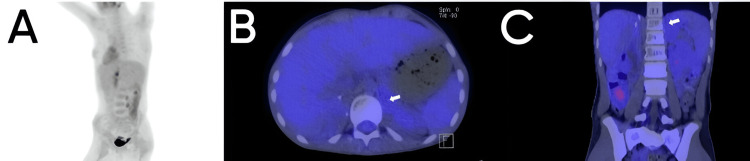
Follow-up with positron emission tomography with fluorodeoxyglucose (PET-FDG) two months after completion of mitotane treatment. Maximum intensity projection (MIP) volumetric images of PET with FDG (right posterior oblique projection) (A); fusion of CT and PET-FDG in the axial plane (B) and coronal plane (C). There is no increase in FDG uptake in the left adrenal gland in the reconstruction in the MIP volumetric images of PET with FDG (A). In the fusion of CT and PET-FDG, it is confirmed that there is no increase in focal FDG uptake (arrow in B and C).

Twelve months after surgery, mitotane was discontinued due to poor response, and a month later, a new PET/CT scan with 18F-FDG showed persistence of metastases in the right psoas, with no evidence of increased uptake in the previously observed left adrenal gland (Figure 4).

## Discussion

ACC has an incidence of 1 to 2 cases per million and can occur from ages 6 to 80, with a median age of approximately 40 [2,3]. Over half of the cases are functioning tumours with hormonal alterations such as hypercortisolism or virilisation, but they can also present as incidental adrenal masses [1-3]. Tumour size, infiltration of adjacent tissues, lymph node involvement, and distant metastases determine the stage and prognosis of this neoplasm, which has a five-year survival rate ranging from 20% to 80% [3,5]. PET/CT with FDG is useful as a predictor of preoperative malignancy as it allows differentiation between benign and malignant lesions. In ACC, PET/CT complements conventional imaging, especially in diagnosing local recurrence. Physiological FDG uptake in adrenal glands has been determined to be mild (SUVmax of 0.95 to 2.46) and lower than hepatic uptake, with an SUVmax less than 2.5 considered normal and increasing in cases of inflammation or malignancy [2,6]. Treatment of ACC is initially based on surgical resection, with better prognosis for tumours that can be completely resected. In patients with complete resection, disease recurrence can be as high as 80% in the first two years. Hence, radiotherapy over the tumour bed and medical treatment for tumour control and hormone overproduction are common [2,3,7]. Mitotane, a derivative of dichlorodiphenyltrichloroethane, is employed in medical therapy owing to its adrenolytic action and inhibition of steroidogenic enzymes, particularly hydroxylase. Despite its therapeutic utility, mitotane is associated with a spectrum of adverse effects. Predominantly, patients experience gastrointestinal, metabolic, and neurological disturbances, as well as skin rashes and hepatic complications [2,6,7]. Despite reports in the literature of FDG uptake in residual adrenal tissue [2,6], a definitive cause remains elusive, leading to the formulation of several theories. Four hypotheses have been proposed: 1) Compensatory hypertrophy, which may occur soon after surgical resection of the contralateral gland; 2) Uptake of adjacent brown fat that may mimic adrenal uptake; 3) Presence of lesions, particularly metastases, in residual adrenal tissue; and 4) Post-mitotane treatment. Absorption in the remnant adrenal gland can occur early and up to 24 months after adrenalectomy and initiation of mitotane treatment. Normal glandular size and morphology may suggest the presence of healthy glandular tissue rather than tumour recurrence [2,6], as in the case of the patient we are describing, where absorption disappeared one month after discontinuing mitotane. Despite inhibiting adrenal cortisol production, adrenal cortical cells become avid for FDG probably due to adrenocorticotropic hormone stimulation, which remains high or normal despite steroid supplementation [2]. This proposition is supported by the fact that patients with pheochromocytoma do not receive mitotane and show no increase in residual gland uptake [6].

## Conclusions

In conclusion, the presented case underscores the diagnostic and therapeutic challenges associated with ACC. The use of PET/CT with FDG, while valuable in distinguishing benign from malignant lesions and assessing treatment response, can pose interpretative challenges, as demonstrated in this case. ACC remains a rare malignancy with a grim prognosis, highlighting the urgent need for further research into novel diagnostic modalities, treatment strategies, and predictive biomarkers. Despite advancements in imaging and therapeutic approaches, optimal management of ACC remains elusive, necessitating ongoing collaboration between clinicians, researchers, and imaging specialists to improve outcomes for patients affected by this aggressive malignancy. Continued investigation into the underlying mechanisms of FDG uptake in residual adrenal tissue post-adrenalectomy and its correlation with treatment response is crucial for refining diagnostic algorithms and therapeutic interventions in ACC. Ultimately, this case serves as a poignant reminder of the complexities inherent in the management of ACC and underscores the imperative for continued research efforts to advance our understanding and management of this challenging disease entity.
